# SG-APSIC1165: Hand hygiene feedback card—Providing real-time feedback to improve hand hygiene compliance

**DOI:** 10.1017/ash.2023.47

**Published:** 2023-03-16

**Authors:** Malathi Maruthasalamoorthy, Asnah Binte Ibrahim

**Affiliations:** St Luke’s Hospital, Singapore; Singapore, St Luke’s Hospital, Singapore

## Abstract

**Objectives:** Hand hygiene is widely recognized as the most effective practice for preventing healthcare-associated infections. Despite ongoing interventions and strategies implemented by the infection control committee, the compliance with and consistency in the hand hygiene practice remains a challenge. At times, staff are unaware when they are noncompliant with hand hygiene; therefore, a more robust strategic approach is needed. One such strategy is customized audit with real-time feedback. A literature review highlighted the effectiveness of audit coupled with specific feedback. This approach was also supported by several guidelines and regulatory bodies that recognized the importance of audit and feedback in hand hygiene improvement efforts. For example, the World Health Organization (WHO) has emphasized 5 core components of improving hand hygiene. One of these components is evaluation and feedback. We sought to provide feedback to healthcare personnel when they do not show compliance with the Five Moments of Hand Hygiene. We aimed to achieve >95% hand hygiene compliance among healthcare staff. **Methods:** Information on the use of the hand hygiene feedback card was provided to the auditors. The hand hygiene feedback card procedure began in all the wards in May 2020. This process first started with orientation of the auditors regarding the hand hygiene feedback card, followed by auditing hand hygiene practice. Staff who did not comply with hand hygiene procedures were given real-time feedback via a card that specified the missed hand hygiene movement. **Results:** Overall hand hygiene compliance among healthcare staff increased by 6% after the hand hygiene feedback card procedure was implemented. **Conclusions:** Overall, the hand hygiene feedback card was effective in improving hand hygiene. Through this quality improvement project, significant and sustained gains in hand hygiene compliance rates of >95% can be achieved.

